# Circulating HPV16 DNA may complement imaging assessment of early treatment efficacy in patients with HPV-positive oropharyngeal cancer

**DOI:** 10.1186/s12967-020-02330-y

**Published:** 2020-04-15

**Authors:** Tomasz W. Rutkowski, Agnieszka M. Mazurek, Mirosław Śnietura, Beata Hejduk, Maja Jędrzejewska, Barbara Bobek-Billewicz, Andrea d’Amico, Wojciech Pigłowski, Andrzej Wygoda, Krzysztof Składowski, Zofia Kołosza, Piotr Widłak

**Affiliations:** 1 I Radiotherapy and Chemotherapy Clinic, Maria Sklodowska-Curie National Research Institute of Oncology Gliwice Branch, Wybrzeze Armii Krajowej 15, 44-101 Gliwice, Poland; 2 Center for Translational Research and Molecular Biology of Cancer, Maria Sklodowska-Curie National Research Institute of Oncology Gliwice Branch, Wybrzeze Armii Krajowej 15, 44-101 Gliwice, Poland; 3 Tumor Pathology Department, Maria Sklodowska-Curie National Research Institute of Oncology Gliwice Branch, Wybrzeze Armii Krajowej 15, 44-101 Gliwice, Poland; 4 Department of Radiology and Diagnostic Imaging, Maria Sklodowska-Curie National Research Institute of Oncology Gliwice Branch, Wybrzeze Armii Krajowej 15, 44-101 Gliwice, Poland; 5 Department of PET Diagnostic, Maria Sklodowska-Curie National Research Institute of Oncology Gliwice Branch, Wybrzeze Armii Krajowej 15, 44-101 Gliwice, Poland; 6 Department of Biostatistic, Maria Sklodowska-Curie National Research Institute of Oncology Gliwice Branch, Wybrzeze Armii Krajowej 15, 44-101 Gliwice, Poland

**Keywords:** Oropharyngeal cancer, HPV, Radiotherapy, Follow-up, Treatment failure, Salvage

## Abstract

**Background:**

Early detection of treatment failure may improve clinical outcome and overall survival in patients with head and neck cancer after first-line treatment. Circulating cell-free HPV16 DNA (cfHPV16 DNA) was evaluated as a possible complementary marker to radiological assessment of early response in patients with HPV-related oropharyngeal cancer (OPC) after radiotherapy alone or combined with chemotherapy.

**Methods:**

The study included 66 patients with HPV-related OPC receiving radical radiotherapy alone or in combination with chemotherapy. cfHPV16 DNA was assessed in the blood of all patients before treatment using TaqMan-based qPCR. Subsequent analysis of cfHPV16 DNA was performed 12 weeks after treatment completion, along with radiological assessment of early treatment results.

**Results:**

Complete (CRR) and incomplete radiological response (IRR) was found in 43 (65%) and 23 (35%) patients respectively. cfHPV16 DNA was present in 5 (28%) patients with IRR, while only in 1 (4%) with CRR. Three of five patients with IRR that were positive for cfHPV16 DNA exhibited histopathologically confirmed local or regional treatment failure, and other two developed distant metastases. None of the patients with negative cfHPV16 DNA presented disease failure.

**Conclusion:**

The post-treatment assessment of cfHPV16 DNA in patients with HPV-related OPC may be used as a complementary biomarker to conventional imaging-based examinations for early identification of treatment failure.

## Background

Radiotherapy (RT) alone or in combination with cisplatin-based chemotherapy (CHRT) is the standard treatment for patients with advanced head and neck cancer (HNC) [1]. Surgical salvage is the treatment of choice both in the case of residual disease after RT/CHRT and in the case of locoregional recurrence. The follow-up visits include periodic physical examination of head and neck region, flexible endoscopy, computerized tomography (CT), magnetic resonance imaging (MRI) or 18F-Fluorodeoxyglucose positron emission tomography (^18^F-FDG PET). However, there are controversies regarding the optimal frequency of follow-up visits and control diagnostic imaging. Nevertheless, it is assumed that the sooner treatment failure is detected the better are the results of salvage [2].

Traditionally, surgical salvage treatment is advised following a clinical examination and imaging performed 10–12 weeks after treatment, but such early interpretation could be difficult due to treatment-related changes that limit radiological evidence of residual disease [3]. Thus, early biomarkers of early and late treatment results could be useful in this clinical setting. Unfortunately, no validated biomarkers are available for head and neck cancers, in particular for those associated with heavy tobacco use and alcohol consumption. The emergence of HPV-driven OPC (human papillomavirus driven oropharyngeal cancer) has opened up new possibilities. As these cancers are a consequence of a viral infection, their molecular profile is distinct from those induced by tobacco and alcohol abuse. Contrary to HPV-negative tumors, where carcinogenesis is enabled by genetic alterations in HPV-associated cancers viral proteins interacting with cellular regulators are necessary to drive HPV-associated carcinogenesis. Due to this, mutations are less frequently found in HPV-associated cancers. Also the concept of field cancerization seems to be more specific for HPV-negative tumors whereas for HPV-associated tumors HPV infection is the starting event [4].

Recent studies showed that circulating cell-free HPV16 DNA (cfHPV DNA) could be found in the blood of most patients with HPV-related OPC and that the amount of cfHPV16 DNA changes according to the treatment response [5–7]. However, very few studies have examined the relationship between plasma levels of cfHPV16 DNA and radiological response in patients with HPV-related OPC shortly after treatment. The aim of this study is to evaluate the role of cfHPV16 DNA as a complementary marker for radiological assessment of treatment results in patients with HPV-related OPC. In day-to-day clinical practice, we can combine results of two independent tests to be more confident of the diagnosis.

## Materials and methods

### Patients

A total of 216 new diagnosed patients with histologically confirmed OPC, who had been admitted to the I Radioteherapy and Chemotherapy Clinic at Maria Sklodowska-Curie National Research Institute of Oncology in Gliwice between September 2012 and September 2016 were included in the study. Patients with metastatic disease or immune suppression were not eligible. At the time of the study recruitment (presentation to the institution), informed consent was obtained from all the patients. The project was approved by the Bioethics Committee at Maria Sklodowska-Curie National Research Institute of Oncology. The study conforms to the Code of Ethics of the World Medical Association. Plasma was obtained from the patients for cfHPV16 DNA assessment prior to any treatment. In those patients who appeared to be HPV positive, serial cfHPV16 DNA blood samples were collected at the time of treatment completion and about 12 weeks later. At that time the response was also assessed by clinical examination and ^18^F-FDG PET-CT, CT, or MRI.

Only patients who underwent definitive treatment [RT alone or RT combined with chemotherapy (CHT)] were included in the study. RT was delivered for over 7 weeks by incorporating five fractions per week combined with CHT (cisplatin, 100 mg/m^2^ days 1, 22, 43) or as a concomitant boost (CB) with seven fractions per week without chemotherapy. Clinical target volume 1 (CTV1) included a primary tumor and involved lymph node groups. CTV2 included areas at risk of harboring microscopic disease and elective lymph node groups. All patients were treated with doses of 70 Gy in 35 fractions (2.0 Gy/fraction) for over 7 weeks or 70.2 Gy in 39 fractions (1.8 Gy/fraction) for over 5.5 weeks to the primary target. Doses to the elective target were 50 Gy in 25 fractions (2.0 Gy/fraction) or 54 Gy in 30 fractions (1.8 Gy/fraction). Induction chemotherapy consisted of 2–3 cycles of TPF (docetaxel 75 mg/m^2^, cisplatin 75 mg/m^2^, d1 and 5-fluorouracil 750 mg/m^2^ d1–5) or PF (cisplatin 100 mg/m^2^, d1 and 5-fluorouracil 1000 mg/m^2^ d1–5).

All patients were followed-up periodically with the first visit after about 12 weeks after treatment and thereafter each 3 months for the 1st year and each 6 months later on or more often if necessary. In the 12 week after treatment (median 12, range 10–16 weeks), MRI, CT, or ^18^F-FDG PET-CT was performed for early treatment results assessment. MRI was performed for 19 patients (30%), CT for 34 patients (52%) and ^18^F-FDG PET-CT for 13 patients (18%). To assess the value of cfHPV DNA as a complementary marker to conventional imaging-based examination, the sensitivity, specificity, positive prognostic value (PPV) and negative prognostic value (NPV) of imaging examination alone and supplemented with cfHPV DNA were calculated. In this study, the term cfHPVrem referred to the complete molecular remission of cHPV16 DNA in plasma after the treatment.

### Plasma sample collection and DNA extraction

Peripheral blood (12 ml) was collected into K_3_EDTA tubes (Becton–Dickinson, New Jersey, Franklin Lakes, USA). Plasma was separated within an hour by double centrifugation at 300×*g* and 1000×*g*, both at 4 °C for 10 min. Aliquots of the plasma were frozen at − 80 °C. DNA was extracted (according to the manufacturer’s instructions) from 1 ml of plasma by the Genomic Mini AX Body Fluids kit (A&A Biotechnology, Gdynia, Poland).

### Analysis of cfHPV16 DNA in plasma

For measurement of the total circulating cell-free DNA in blood (cfDNA), the amplification of TERT (human telomerase reverse transcriptase) was used as described previously [8]. Shortly, each measurement consisted of a standard curve, negative control and a sample. For the construction of the standard curve, we used a control genomic DNA. The concentration of the total cfHPV DNA did not influence the cfHPV DNA detection as we presented before [8]. For HPV16 detection, independent reaction was performed using primers and probe set for HPV16 genome. Each measurement consisted of a standard curve, negative control and a sample. A standard curve using tenfold DNA dilutions of plasmid construct containing HPV16 genome was plotted. The obtained copies of cfHPV DNA were calculated according to the amount of plasma that was taken for DNA extraction (copies/ml). PCR reactions were performed using the Bio-Rad CFX96 qPCR instrument (Bio-Rad Laboratories, Hemel Hempstead, United Kingdom). If HPV16 was found, it’s presence would be confirmed with a second independent DNA isolation.

### Tumor samples: detection of HPV and confirmation of its biological activity in tumor tissue

Detection of biologically active HPV in the tumor samples was performed following a previously described two-stage procedure [9]. In the first step, DNA of high oncogenic risk HPV was detected by means of real time PCR. Thereafter, its transcriptional activity was confirmed by immunohistochemistry demonstrating the accumulation of P16(INK4A) protein in the tumor tissue.

Tumor DNA was isolated from archival paraffin-embedded tissue samples (FFPE) available for 66 cases. For each block, from three to five 10-μm-thick sections were collected in an aseptic manner and processed in a MagCore HF16 Plus automated nucleic acid extractor using No 405 Genomic DNA FFPE One-Step Kit (RBC Bioscience Corp., Ne Taipei City, Taiwan). The DNA purity and concentration were evaluated using a DS-11FX spectrophotometer (DeNovix, USA). Detection of HPV in FFPE tumor samples was made using a RealTime High Risk HPV test (Abbott Molecular, Abbott Park, Illinois, USA) which can detect 14 HR-HPV types in the same reaction and can also differentiate between HPV16, HPV18, and 12 other less common HPV types. Usefulness of the test to assess HPV status in formalin-fixed, paraffin-embedded tissue samples was demonstrated elsewhere [9–11]. On completion of sample preparation, a reaction mixture containing amplification master mix and sample solution containing 25 ng of extracted DNA was prepared according to the manufacturer’s instructions (Abbott Molecular, Abbott Park, Illinois, USA). Thermocycling and product detection were performed using a ViiA 7 real-time instrument (ThermoFisher Scientific). Samples for which the β-globin Ct value > 36 were considered to be non-informative. Negative and positive controls were included in each run to verify that sample processing, amplification, and detection steps were performed correctly. The negative control was formulated with DNA containing a β-globin sequence and poly-dA:dT as carrier DNA. The positive control contained HPV16, HPV18, HPV58, and β-globin sequences tied to the carrier DNA.

To confirm HPV biological activity, P16(INK4A) expression in tumor tissue was demonstrated by immunohistochemistry using E6H4 monoclonal antibody and Benchmark Ultra staining system (CINtec p16 Histology kit, Ventana Medical Systems Inc., Tucson, USA), according to the manufacturer’s instructions. Only patients with uniform, strong staining of at least 80% of tumor cells were classified as positive. Focal expression of the protein in single cells or limited groups of cells, especially in the basal layer of the epithelium, was regarded as negative.

## Results

### Patients and tumor characteristics according to cfHPV DNA

In the present study, all the 216 OPC patients were Caucasians. Tissue samples were not available for 95 out of 216 patients (44%) and noninformative for 5 patients (2%) due to missed cancer tissue after previous histopathological assessment. HPV16 DNA testing of tumor biopsies was negative in 30 out of 116 (25.9%) and positive in 86 out of 116 (74.1%) cases. The HPV type 16 was the most common genotype with a frequency of 95.3% of all infections. Prior to any treatment, circulating cell-free HPV16 DNA was found in 82 (38%) out of 216 patients. Eight patients underwent palliative treatment, one patient had a virus type other than HPV16, two patients were lost from follow-up controls and therefore these individuals were not included in the study.

Finally, 66 cfHPV-positive OPC patients were included in the present study. The median of cfHPV16 DNA viral load of those 66 patients was 1152 copies/ml (136 l.q.–7723 u.q.). Table 1 summarizes the level of pre-treatment cfHPV16 DNA viral load depending on clinical parameters. Statistical analysis (Mann–Whitney test) revealed that significantly lower cfHPV16 DNA viral load was observed for patients with T1 tumor compared to those with T2 (p = 0.038), T3 (p = 0.006) or T4 (p = 0.019). No correlation was found for cigarette consumption nor N classification.

**Table 1:** Patient and tumor characteristics for the analyzed group

Clinical parameter	No of cases 66 (%)	Pre-treatment viral load median (u.q.–l.q) [copies/ml]	cfHPV16 DNA in the 12th week		*p* value
			Positive No of cases (%)	Negative No of cases (%)	
Tumor classification					
T1	14 (21)	76 (25–177)	0 (0)	14 (23)	0.327
T2	18 (27)	1422 (191–7913)	1 (17)	17 (28)	
T3	19 (29)	2874 (203–12,320)	2 (33)	17 (28)	
T4	15 (23)	1548 (176–5239)	3 (50)	12 (20)	
Nodal classification					
p	6 (9)	61 (21–229)	0 (0)	6 (10)	0.232
N0–N1	8 (12)	1283 (252–3607)	2 (330	6 (10)	
N2–N3	52 (79)	1489 (140–9092)	4 (67)	48 (80)	
Treatment					
RT	6 (9)	1722 (229–2022)	0 (0)	6 (10)	0.405
CH-RT	9 (14)	7913 (167–13,412)	2 (33)	7 (12)	
CH-CHRT	30 (45)	2071 (163–10,523)	3 (50)	27 (45)	
CHRT	21 (32)	146 (36–1152)	1 (17)	20 (33)	
Cigarette consumption					
Never	33 (50)	545 (101–7913)	3 (50)	30 (50)	0.734
≥ 5 years	10 (15)	1722 (163–23,008)	0 (0)	10 (17)	
Current smokers	23 (35)	1396 (176–5600)	3 (50)	20 (33)	

*p* post resection, *RT* radiotherapy, *CH-RT* induction chemotherapy, *CH-CHRT* induction chemotherapy followed by radiochemotherapy, *CHRT* radiochemotherapy, *l.q.* lower quartile, *u.q.* upper quartile

The group consisted of 39 (59%) men and 27 (41%) women in a mean age of 55 years (range: 30–75 years). All but one patient (T2N0) presented advanced disease (IV stage according to AJCC 7th edition). In 7 (11%) patients, metastatic neck lymph nodes had been dissected as a diagnostic procedure prior to presentation at the I Radiotherapy and Chemotherapy Clinic. To find out if there is any relationship between main clinical factors and cfHPV16 DNA, correlation between stage of disease (T or N status), treatment strategy or cigarette consumption and probability of cfHPV16 DNA detection in the 12th week was assessed. Results are presented in Table 1.

### Radiological and molecular response in the 12th week

Radiological response of treatment in about 12th week (median 12, range 10–16 week) was assessed by ^18^F-FDG PET-CT, CT or MRI. Complete radiological response (CRR) was defined as disappearance of all signs of cancer in imaging in response to treatment in the 12th week. Incomplete radiological response (IRR) was defined as the presence of residual cancer signs in imaging at that time. Molecular responses ware quantified by measuring the reduction in cfHPV16 DNA relative to an initial quantity. The complete cfHPV16 DNA remission was defined as disappearance of cfHPV16 DNA in blood (cfHPV16rem) after treatment. Molecular cfHPV16 DNA recurrence was defined as a cfHPV16 DNA appearance after cfHPV16 DNArem (cfHPV16rec).

In the 12th week after RT/CHRT 43 patients (65%) achieved a CRR and 23 (35%) achieved an IRR. The molecular remission of cfHPV16 DNA had 60 patients (91%, cfHPV16rem) and in 6 (9%) patients cfHPV16 DNA was still present in the blood at that time.

Among 23 patients who were qualified as IRR in the 12th week, 18 (27%) patients had cfHPV16rem and in 5 (8%) patients cfHPV16 DNA was still present in the blood at that time. Among 43 patients who were qualified as CRR in the 12th week, 42 (64%) patients had cfHPV16rem and in 1 (1%) patient cfHPV16 DNA was still present in the blood at that time. Thus, in the 12th week the concordance of CRR with complete cfHPV16 remission was 64%, the concordance of IRR with cfHPV16 DNA still presented in the blood was 8%, giving total compliance 72%. Results mismatch was at 28% (19/66).

### Complete radiological response—follow-up during next 6 months

12 weeks after RT/CHRT, 43 (65%) patients had CRR. In 1 patient, cfHPV16 DNA was detectable at that time despite no radiological signs of active disease. During follow-up, cfHPV16 DNA remission was found in this patient after the next 3 months (patient: #29, Table 2) and no evidence of disease was found in the other from this group.

**Table 2:** Patients with incomplete radiological response 12 weeks after treatment (additional patient (29) with complete radiological response but with positive cfHPV16 DNA)

Patient no.^a^	12 weeks after treatment			Subsequent (> 12 weeks)			Final result
	Radiol. finding	cfHPV16 DNA	Intervention (pathology)	Radiol. finding	cfHPV16 DNA	Intervention (pathology)	
1 (3)	L	−	−	NED	−	−	Cured
2 (4)	N	−	ND (−)	NED	−	−	Cured
3 (5)	N	−	−	NED	−	−	Cured
4 (10)	N	+	−	Liver	+	Biopsy (+)	Distant (liver)
5 (11)	L + N	−	−	NED	−	−	Cured
6 (17)	N	−	Biopsy (−)	NED	−	−	Cured
7 (19)	L	+	Tumorectomy (+)	L	+	Biopsy (+)	Local failure
8 (20)	N	+	ND (+)	Lung	+	−	Distant (lung)
9 (22)	N	+	ND (+)	NED	−	−	Cured
10 (24)	L	−	−	NED	−	−	Cured
11 (29)	−	+	−	NED	−	−	Cured
12 (31)	N	−	ND (−)	NED	−	−	Cured
13 (40)	N	−	−	NED	−	−	Cured
14 (42)	N	−	−	NED	−	−	Cured
15 (44)	N	−	Biopsy (−)	NED	−	−	Cured
16 (46)	N	−	−	NED	−	−	Cured
17 (48)	N	−	Biopsy (−)	NED	−	−	Cured
18 (53)	N	−	−	NED	−	−	Cured
19 (54)	N	−	Biopsy (−)	NED	−	−	Cured
20 (55)	N	−	Biopsy (−)	NED	−	−	Cured
21 (58)	N	−	−	NED	−	−	Cured
22 (59)	N	−	−	NED	−	−	Cured
23 (63)	N	−	−	NED	−	−	Cured
24 (66)	N^b^	+	−	Lung	+	−	Distant (lung)

*L* local residual disease, *N* nodal residual disease, *ND* nodal dissection, *NED* no evidence of disease

^a^In brackets numbers of consecutive patients as discussed in text

^b^Mediastinal nodes, radiol. finding—result of PET or MR or TK—first radiological assessment after treatment (12 weeks after treatment)

### Incomplete radiological response—follow-up during next 6 months

12 weeks after RT/CHRT, 23 (35%) patients had incomplete radiological response. Residual, nodal or local disease was detected radiologically in 19 and 3 patients, respectively. Residual, both, nodal and local disease was found in 1 patient.

Of all 23 patients with IRR, 5 had cfHPV16 DNA still present in the blood (patients: #10, #19, #20, #22, #66, Table 2), and 18 had cfHPV16rem. Three patients (patients: #19, #20 and #22, Table 2) of these 5 with still detectable cfHPV16 DNA underwent salvage dissection with residual cancer confirmation in postsurgical histopathological specimens, which showed squamous cell carcinoma and p16 expression. Follow up after the first 12 weeks from treatment termination lasting next 6 months, showed that in one patient cfHPV16 DNA was still detected after successful salvage, and subsequent PET examination revealed metastatic lung tumor (patient #20, Table 2). Second patient underwent a surgical salvage for local failure yet the treatment was not effective, and was accompanied by a persistence of cfHPV DNA in the blood and disease progression (patient #19, Table 2). In the third patient lymph node dissection was successful, cfHPV16 DNA was not detected latter on during follow-up and no signs of cancer was observed (patient #22, Table 2). Another patient with partial cfHPV16 DNA remission from the IRR group demonstrated increased ^18^F-FDG uptake in the liver, with no uptake at the primary site or cervical lymph nodes (patient: #10, Table 2). Core biopsy of the liver tumor confirmed metastatic p16 positive squamous cell carcinoma in this patient. The fifth patient demonstrated increased ^18^F-FDG uptake in mediastinal lymph nodes but due to the size and the level of uptake the nodes were found to be not metastatic at that moment, but metastatic disease to lung and mediastinal nodes became obvious in subsequent ^18^F-FDG PET-CT (patient: #66, Table 2).

**Fig. 1:**
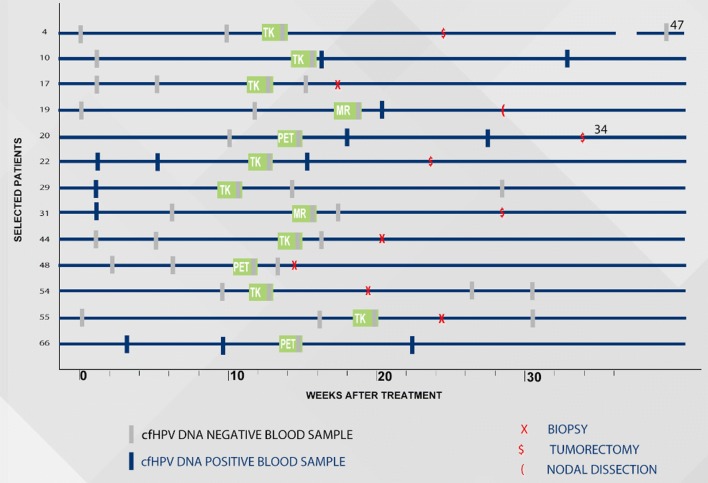
The timeline for selected patients from IRR group who underwent intervention due to incomplete radiological response 12 weeks after treatment completion and/or presented cfHPV16 DNA at that time

Of the 18 patients with cfHPV16rem in IRR group, 5 patients underwent a biopsy from the suspected sites, but results failed to demonstrate residual tumor (patients: #17, #44, #48, #54, #55, Table 2). In 2 patients, lymph node dissection was performed, but their specimens showed no evidence of cancer (patients #4 and #31, Table 2). The remaining 11 patients underwent radiological and molecular observation. Finally, in all 18 patients (7 after biopsy or lymph node dissection, and remaining 11 patients without any intervention), consecutive radiological assessment performed in the subsequent 3 months revealed complete radiological response (and no cfHPV16 DNA in blood as well). Figure 1 presents the timeline for selected patients from IRR group to better visualize clinical situation for these with positive cfHPV16 DNA at 12 weeks and for these who underwent surgical intervention (Fig. 1).

### Specificity and sensitivity of detecting treatment failure based on cfHPV16 DNA assessment

In subsequent imaging examinations (i.e. after 12 weeks), complete remission of disease was found in all 62 (CRR + IRR) patients with cfHPVrem, after exclusion of the patient who was not cured with the salvage (patient: 19, Table 2), one after successful salvage but with subsequent lung metastases (patient: 20), one patient with liver metastases (patient: 10, Table 2), and one patient with lung metastases (patient: 66, Table 2) in whom HPV16 DNA was continuously detectable. All but one patient with detectable HPV16 DNA 12 weeks after treatment completion (patient: 29, Table 2) finally developed treatment failure (local, regional or distant) giving sensitivity and specificity of the tested method to predict treatment failure generally of 100% and 98% respectively and PPV and NPV of 83% and 100% respectively (Table 3).

**Table 3:** Specificity and sensitivity of the detection of treatment failure based on cfHPV DNA assessment exclusively

cfHPV16 DNA	Treatment failure (residual or metastatic disease) No of individuals		
	Residual or metastatic disease	Cured	Total
Positive	5^a^	1^b^	6 (PPV, 83%)
Negative	0	60	60 (NPV, 100%)
Total	5 (sensitivity, 100%)	61 (specificity, 98%)	66

*NPV* negative predictive value, *PPV* positive predictive value

^a^Patients: 10, 19, 20, 22, 66

^b^Patient: 29

## Discussion

Locoregional treatment failure is one of the main reasons for poor RT or CHRT results in patients with advanced HNC. Long-term follow-up in patients treated for HNC is routinely performed to detect persistent disease, recurrence, metastasis or second primary tumors at the earliest opportunity. However, due to treatment-related changes imaging based estimation seems to be inconclusive until 12–14 weeks after the treatment. About a third of patients present incomplete imaging response at this time [12].

In cases of clinical partial nodal response about 60% [13–16] of neck dissection specimens did not contain viable tumor. Moreover, ultrasound-guided fine needle aspiration cytology is barely helpful in defining candidates for ND after RT/CHRT with specificity as low as 42% [14]. One should remember that ND is not neutral for patients after RT/CHRT and may cause significant morbidity both on the neck and at the primary site (increase of fibrosis, swallowing problems, delayed mucosal healing and patient anxiety) [17]. Due to this, new markers are sought to support decisions about salvage surgery in this population of patients. Despite some promising data [18], no reliable serum tumor markers are currently available in clinical practice to indicate those patients with residual disease [3]. Due to HNC biological diversity, the identification of a molecular signature that fits all tumors universally is precluded, but HPV-related HNC may be the exception.

Due to causal association between HPV and OPC, the previous dogma as to the epidemiology, pathology, and clinical course of HNC has been revised [19]. Although the prognosis of patients with HPV-driven OPC is significantly better than that of their HPV-negative counterparts, up to 25% of patients will recur within 24 months of treatment [20, 21]. In contrast to the traditionally poor prognosis of patients with recurrent OPC, the outcomes of such patients with HPV-related OPC have improved considerably over the last 2 decades, now resulting in overall survival of up to 50% over 5 years [22]. Moreover, it is estimated that the incidence of HPV-related OPC will increase in the next few years and will likely constitute a majority (approximately 47%) of all head and neck cancers by 2030 [23].

Circulating cell-free HPV16 DNA is found in blood of most patients with HPV-related OPC. The observation that the concentration of HPV16 DNA is changing during the treatment of patients with HPV related OPC is not new [5–7]; however, most of the data is derived from retrospective studies with obvious limitations, including small cohorts, lack of post-treatment surveillance and post-treatment HPV16 DNA assessment. Cao et al. observed a gradual decline in HPV16 DNA during RT that became undetectable in all patients with HPV-related OPC at the end of treatment. There were also no obvious differences in the rate of HPV16 DNA decline between the patients with eventual tumor relapse and those without [5]. Ahn et al. [24] reported that from 35 patients with HPV-16–positive pretreatment plasma, cfHPVrem was found after treatment completion in 30 of them (86%). Moreover, Lee et al. [25] reported HPV16 DNA level below the threshold of detection in 96% of patients with OPC after CHRT and HPV16 DNA detectable prior to the treatment. Mazurek et al. [8] found a decreased level of cfHPV16 DNA during therapy, which became undetectable on the last day of therapy in all patients. These observations indicate that the amount of cfHPV16 DNA decreases during RT or CHRT is usually becoming undetectable at the end of successful treatment. Moreover, detectable HPV16 DNA in the blood of patients after the treatment seems to increase the risk of disease recurrence. Ahn et al. described a significantly higher risk of the treatment recurrence in patients with HPV16 DNA present in the blood after treatment after adjusting for alcohol use, T classification, N classification and smoking status. No correlation with residual disease was done, but 5 patients exhibited HPV16 in post-treatment plasma samples, and 4 of these went on to develop recurrence during further follow-up time [24].

However, no correlation of post-treatment HPV16 DNA status and radiological response was performed in the abovementioned studies [5, 7, 8, 24]. More detailed information could be drawn from Lee et al. who focused on predicting response to RT/CHRT basing on circulating HPV16 DNA after treatment, correlating results with results based on ^18^F-FDG PET-CT. Lee et al. found that despite an increased ^18^F-FDG uptake in few patients, there was no treatment failure if cfHPVrem was observed. Biopsies from the PET-avid sites failed to demonstrate residual tumor and in all further ^18^F-FDG PET-CT scans demonstrated complete resolution of disease. Nodal failure was presented in one patient with continuously elevated HPV16 DNA with increased ^18^F-FDG uptake [25].

In our cohort, incomplete radiological response was found in 35% of patients, mostly in regional nodes. Only 5 patients (28%) had residual HPV16 DNA in the blood at that time. Treatment failure was confirmed histopathologically only in these patients. All others with incomplete radiological response, but with cfHPVrem had complete resolution of the disease in subsequent examination. This observation suggests that cfHPV16 DNA testing along with the imaging may be more precise than imaging alone in indicating patients with residual disease after RT/CHRT.

Few of our patients with residual neck mass despite of cfHPVrem underwent ND or biopsy, and no cancer tissue was not confirmed in any of them. Similarly, Lee et al. showed that those who underwent ND due to ^18^F-FDG uptake in cervical nodes but with cfHPVrem failed to show any histopathologically proven residual tumor tissue [25].

Our results showed that tracking of cfHPV16 DNA in patients with HPV related OPC shortly after RT or CHRT may help identify residual disease both at the primary site and in regional nodes that were not evident from radiographic imaging. In such patients cfHPV16 DNA testing complements imaging-based assessment and considerably increases the specificity and PPV of failure detection, which may enhance the clinical decision-making process regarding surgical approach. In patients with cfHPVrem, postponing surgery could be reasonable, while it seems inevitably necessary in those with cfHPV16 DNA present in the blood.

Moreover, we hypothesize that the detectable HPV16 DNA several weeks after treatment is not only a sign of an active HPV-related cancer as a residual disease, but also may reflect the presence of disease outside of the treated region. In two of our patients with IRR and elevated cHPV16 DNA, subsequent ^18^F-FDG PET-CT revealed metastatic disease. Due to the continued cHPV16 DNA assessment, Lee et al. [25] were able to diagnose the presence of recurrent neck disease 8 months following liver resection due to metastasis. Cao et al. reported a patient who had detectable cfHPV16 DNA in the blood 4 months prior to the detection of lung metastasis. A surveillance chest CT 4 months later revealed a new lung nodule that proved to be a HPV(+) metastatic squamous cell carcinoma on biopsy [5]. Considering these observations, if cfHPV16 DNA is still present in the blood a few weeks after RT/CHRT one should consider ^18^F-FDG PET-CT examination due to the risk of metastasis.

Our findings should be treated with caution as they are preliminary and need to be confirmed in a larger group of patients with a longer surveillance period. At present the sole information about cfHPV16 DNA presence in the blood after treatment should not influence the treatment decision yet unless radiological or clinical suspicion of treatment failure is also found. Nevertheless, the information about cfHPV16 DNA presence should significantly influence the decision to increase follow-up and radiological assessment in this group of patients. The positive correlation of viral load between plasma and tissue, shown in our previous studies [26], strongly confirms the analytical importance of measuring the circulating HPV16 DNA in the plasma. Our current priority is diagnostic validation of qPCR methodology and carrying out scientific research of clinical meaningful of HPV16 DNA viral load in therapy.

## Conclusion

The main conclusion of the article is that cfHPV16 DNA when tested along with radiological assessment may help to differentiate between true residual disease and treatment related changes early enough to decide about salvage in patients after first line treatment due to HPV related OPC. In the era when new possibilities of surgical salvage, stereotactic reradiation or even second line systemic treatment which includes immunotherapy are available, early diagnose of treatment failure seems to be crucial to give the patient another chance. Moreover, regular assessment of HPV16 DNA during follow-up may help to detect not only the recurrence, but also distant metastases prior to onset of clinical symptoms, allowing for more effective salvage therapy. The correlation between HPV16 DNA detection in the blood and results of RT/CHRT is particularly interesting due to the anticipated increase in patients suffering from HPV-related OPC and significantly longer survival despite the recurrence. Results should be treated with caution, as a preliminary but of significant practical meaning that may turn into clinical benefit for patients with OPC.

## Data Availability

The authors declare that all original data are available for inspection and evaluation.

## Abbreviations

HPV: Human papillomavirus
DNA: Deoxyribonucleic acid
cfHPV16 DNA: Circulating cell-free HPV16 DNA
cfHPV16 DNArem: Complete molecular remission of cHPV16 DNA in plasma after the treatment
qPCR: Quantitative polymerase chain reaction
FFPE: Paraffin-embedded tissue samples
HNC: Head and neck cancer
OPC: Oropharyngeal cancer
ND: Nodal dissection
CRR: Complete radiological response
IRR: Incomplete radiological response
PPV: Positive prognostic value
NPV: Negative prognostic value
RT: Radiotherapy
CHRT: Chemoradiotherapy
CT: Computerized tomography
MRI: Magnetic resonance imaging
^18^F-FDG PET: 18F-Fluorodeoxyglucose positron emission tomography
AJCC: The American Joint Committee on Cancer staging system
PET: Positron emission tomography
CB: Concomitant boost
CTV: Clinical target volume
